# High Temporal Summation of Pain Predicts Immediate Analgesic Effect of Acupuncture in Chronic Pain Patients—A Prospective Cohort Study

**DOI:** 10.3389/fnins.2019.00498

**Published:** 2019-07-11

**Authors:** Petra Iris Baeumler, Peter Conzen, Dominik Irnich

**Affiliations:** ^1^Multidisciplinary Pain Center, Department of Anaesthesiology, University Hospital Ludwig-Maximilians-University, Munich, Germany; ^2^Department of Anaesthesiology, University Hospital Ludwig-Maximilians-University, Munich, Germany

**Keywords:** quantitative sensory testing, wind-up ratio, vibration detection threshold, responder, sensitization

## Abstract

**Objectives:** This prospective cohort study explored whether two distinguished sensory parameters predicted acupuncture effects in chronic pain patients; namely high temporal summation of pain (TS) indicating spinal synaptic facilitation as well as a low vibration detection threshold (VDT) indicating a loss of Aβ-fiber function.

**Methods:** Pinprick induced TS and VDT were assessed by standardized, validated methods at the most painful body site and a pain free control site in 100 chronic pain patients receiving six acupuncture sessions as part of an interdisciplinary multimodal pain treatment (IMPT). Immediate change in pain intensity after the first acupuncture session (first treatment on the first day of IMPT) was assessed by the verbal rating scale (VRS, 0–100). After 4 weeks of treatment, patients indicated in a questionnaire whether acupuncture had relieved pain immediately and whether it had contributed to overall pain reduction and well-being after IMPT.

**Results:** Logistic regression analysis revealed an association between high TS at the control site and a reduction in pain intensity of at least 30% (VRS) after the first acupuncture (OR [95%-CI] 4.3 [1.6–11.8]). Questionnaire ratings of immediate pain relief after acupuncture were associated with high TS at the control site (OR [95%-CI] 3.8 [1.4–10.2] any pain relief, OR [95%-CI] 5.5 [1.7–17.1] over 50% pain reduction) and at the pain site (OR [95%-CI] 3.2 [1.2–8.9] any pain relief). Appraisals of the contribution of acupuncture to overall pain reduction and well-being after IMPT were not associated with TS. The VDT was not associated with any outcome.

**Conclusion:** This explorative study provides first-time evidence that high TS, especially at a pain free control site, but not VDT, might predict immediate analgesic response to acupuncture in chronic pain patients. Thus, highly centrally sensitized chronic pain patients might respond particularly well to acupuncture.

## Introduction

In the management of chronic pain it is crucial to identify effective treatments in order to minimize the patient's risk of further chronification and to reduce health care costs (Main et al., 2008; Institute of Medicine, 2011). Acupuncture has been shown to be effective in treating chronic pain conditions (MacPherson et al., 2017; Vickers et al., 2018) with responder rates of 50% (Vickers and Linde, 2014), but little is known about predictors of treatment response. The effectiveness of acupuncture is thought to be based among other mechanisms on its potential to reduce central sensitization processes that sustain chronic pain states. This involves a reduction of spinal synaptic facilitation and reestablishment of endogenous pain control (Zhao, 2008; Zhang et al., 2014) up to demodulation of maladaptive functional brain neuroplasticity (Maeda et al., 2017). Thus, it stands to reason that chronic pain patients exhibiting strong central sensitization might respond particularly well to acupuncture.

Quantitative sensory testing has proven to permit conclusions about sensitization mechanisms contributing to chronic pain (Arendt-Nielsen, 2015) and standardized methods have been developed. Those suggested by the German Research Network for Neuropathic Pain (DFNS) are easily applied in clinical practice (Rolke et al., 2006), show good test-retest and interobserver reliability (Geber et al., 2011) and come with the advantage of established reference values (Rolke et al., 2006).

Increased temporal summation of pain (TS) is a sensory sign observed in various chronic pain conditions (Eide et al., 1994; Nikolajsen et al., 1996; Pud et al., 1998; Graven-Nielsen et al., 2000; Staud et al., 2001; List et al., 2008; Edwards et al., 2011; Gerhardt et al., 2015) and considered to reflect advanced spinal synaptic facilitation. TS describes the rise in pain intensity during repetitive nociceptive stimulation and is regarded as the physiological correlate of wind-up (Eide, 2000). This phenomenon is caused by a rapid facilitation of synaptic transmission between nociceptive afferents and spinal projection neurons during repetitive C- and Aδ-fiber activation (Mendell, 1966; Clarke et al., 2002). If spinal synaptic strength is permanently augmented through long-term potentiation (LTP) in chronic pain states, TS was observed to be elevated (Herrero et al., 2000). Furthermore, high TS was found to be associated with further signs of central sensitization such as hyperalgesia (Rabey et al., 2015), number of pain sites (Gerhardt et al., 2015; Vaegter and Graven-Nielsen, 2016) and impaired endogenous pain control (Staud et al., 2001; Lev et al., 2010; Daenen et al., 2014). Thus, the first hypothesis addressed in this study is that high TS is associated with the response to acupuncture in chronic pain patients.

Additionally, considering that acupuncture effects rely on sensory input elicited by the needle stimulus, it can be proposed that patients exhibiting a loss of function of sensory afferents might respond less to acupuncture. In this regard we obtained hints from a previous study in which acupuncture effects on sensory thresholds were assessed by QST in healthy volunteers. This study provided first-time conclusive support for the importance of segmental inhibition for the analgesic effect of electroacupuncture (Baeumler et al., 2015). In contrast, effects of manual acupuncture varied largely. A sub-analysis revealed a particularly poor vibration detection threshold (VDT), indicating a loss of Aβ-fiber function, in volunteers who did not experience a change in heat or pressure pain thresholds in the segment of needle placement after manual acupuncture (Baeumler et al., 2014). Segmental inhibition describes the reduction of nociceptive transmission in the spinal dorsal root through homosegmental A-fiber activation via inhibitory interneurons (Sandkuhler, 1996; Melin et al., 2013). While Aβ-fiber activation seems mainly responsible for an immediate but transient analgesic effect, low intensity Aδ-fiber activation can induce long-term depression of synapses between primary nociceptors and spinal projection neurons (Sandkühler, 2000). In line with these mechanisms as crucial components of the analgesic effects of acupuncture and other naturopathic stimulation therapies (White et al., 2011; Musial et al., 2013) segmental needling represents an integral part of acupuncture practice (Wancura-Kampik, 2012). Thus, the second hypothesis tested in this study is that a loss in vibration detection might be associated with a poor response to acupuncture in chronic pain patients.

In this study, we explored the association of TS and the VDT, as evaluated by DFNS standards, with the immediate analgesic response to acupuncture and with the subjective evaluation of acupuncture effects after a whole treatment in chronic pain patients undergoing multimodal pain therapy.

## Materials and Methods

### Study Design

In this prospective cohort study TS and VDT were assessed as predictors for the analgesic effect of acupuncture. The study population consisted of chronic pain patients who received six to eight acupuncture treatments in the course of a 4-week interdisciplinary multimodal pain treatment (IMPT). Acupuncture was applied as the first treatment modality on the first day; meaning that effects of other therapies were excluded. The percentage change in pain intensity after this first acupuncture treatment was evaluated by the verbal rating scale (VRS). In addition, the subjectively perceived immediate pain relief experienced through acupuncture and its contribution to the overall pain reduction as well as to the general well-being during the whole 4-week treatment period was assessed by a patient questionnaire.

TS and VDT were evaluated at the most painful site (PS) and a pain free control site (CS) before the first acupuncture treatment and were analyzed as predictors for the acupuncture effect. TS and VDT were also assessed at the last day of IMPT and are not subject of this publication.

### Study Setting

Recruitment of study participants was performed among chronic pain patients undergoing a multimodal pain treatment program, the Munich Outpatient Program in Complementary and Alternative Medicine (MOCAM) at the Multidisciplinary Pain Center at the Department of Anesthesiology, University Hospital of the Ludwig-Maximilians University of Munich, Germany. The MOCAM follows the national criteria for IMPT, combining education, physical exercises, relaxation and cognitive behavioral therapy. MOCAM also integrates complementary methods of Traditional Chinese Medicine (acupuncture, qigong) and classical naturopathy (poultices and packs). It is carried out in an outpatient setting. Recruitment period of this study was from 2013, 3rd June to 2015, 23rd February. Data acquisition was completed on 2015, 20th March.

### Patients

Inclusion criteria were age between 18 and 75 years, chronic pain condition (>3 months), command of the German language and written informed consent. General exclusion criteria were malignant, neurodegenerative or chronic inflammatory diseases. Patients suffering from pain in the hands were also excluded, because the dorsum of the dominant hand served as a pain free control site. Patients who had received less than six acupuncture treatments in the course of the MOCAM were retrospectively excluded. Patients were informed about conduct and purpose of the study in oral and written form after the introductory seminar of 75 min on the first day of the multimodal pain treatment program. Inclusion was performed after the subsequent coffee break in case that written informed consent was obtained.

### Acupuncture

The schedule of the MOCAM stipulates that participating chronic pain patients receive eight acupuncture treatments during the 4-week multimodal pain program. Study documentation included the number of treatments that were actually received. For the course of this study acupuncture was scheduled on the first treatment day as the first intervention. Acupuncture in the MOCAM is exclusively performed by experienced medical acupuncturists with at least 200 h of acupuncture training (A-Diploma of the German Medical Acupuncture Association, *DÄGfA*). Treatments are usually performed in a group setting and point localization, needling depth as well as type and strength of needle stimulation are chosen individually. The individual treatment regimens integrate elements of TCM based acupuncture and western acupuncture techniques. Treatments rely on a differentiation of symptoms according to ba gang as well as on differential point selection according to zang-fu in case of internal diseases or according the theory of qi, blood and fluids. In addition, treatment included meridian orientated choice of points according to the site of pain as well as microsystem and trigger points. Five to ten needles are inserted per treatment. Needling depth is on average 0.5–1 cm and needle stimulation is performed by up and down movement as well as twisting of the needle. Acupuncturists attempt to elicit deqi, but this is not mandatory. Electrical or laser stimulation devices are available, but were not used in any of the patients included in this study. With the needles in place patients relax for 20–40 min.

### Outcome Parameters

#### Acupuncture Responder Based on the Reduction in Pain Intensity After the First Acupuncture Treatment

The pain intensity directly before and after the first acupuncture treatment on the first treatment day of the multimodal pain program was evaluated by the verbal rating scale (VRS, 0 = “no pain,” 100 = “worst pain imaginable”). According to recommendations of the US Initiative on Methods, Measurement, and Pain Assessment in Clinical Trials (IMMPACT) (Dworkin et al., 2005) two definitions for clinically relevant pain relief were applied; reduction in pain scores >30 and >50%. A reduction of pain scores of more than 30% has been shown to represent a clinically meaningful pain relief independent from baseline pain (Farrar et al., 2001). Accordingly, percent change in pain intensity was calculated as (VRSpost – VRSpre)/VRSpre ^*^100 to classify patients as acupuncture responders and non-responders. Patients with a reduction in pain intensity of over 30% after the first acupuncture treatment are referred to as Resp30 and those with a reduction in pain intensity of over 50% are referred to as Resp50.

#### Patient Questionnaire on Acupuncture Effects

After the closing seminar on the last day of the multimodal pain treatment program patients were asked to fill in a questionnaire in order to assess the subjective perception of their response to acupuncture. Patients who were not able to attend this post-treatment visit were offered a separate study visit within the next days, at most 2 weeks later. The questionnaire was designed for the purpose of this study in order to assess the patients' subjective appraisal of immediate pain relief achieved through acupuncture, its contribution to the overall pain reduction during the 4 weeks of treatments as well as its contribution to general well-being. Questionnaire items translated into English read as follows:

1. Did you once experience an immediate pain relief after acupuncture treatments? *Yes/No*
2. If yes, by how much was your pain relieved? *By more than 50%?/By less than 50%?*
3. Do you have the impression that your overall pain relief is related to acupuncture? *Yes/No*
4. If yes, by how much was your pain relieved? *By more than 50%?/By less than 50%?*
5. Do you have the impression that acupuncture contributed to your general wellbeing? *Yes/No*
6. How many acupuncture treatments did you receive? *Free text*

### Predictor Assessment

Assessments of TS and VDT were performed by two examiners (PB and a study nurse) during the waiting time before the first acupuncture treatment on the first day of the multimodal pain treatment program. Both sensory signs were determined at the most painful body site (PS) and at the dorsum of the dominant hand, which served as a pain free control site (CS). In headache cases, the temple was classified as the pain site. Measurements were performed according to the protocol for quantitative sensory testing (QST) designed by the German Research Network on Neuropathic Pain (DFNS) (Rolke et al., 2006). According to DFNS reference data, a certain value range at the bottom of the TS scale and at the top of the VDT scale can be regarded as not aberrant. Just exceedingly high TS indicates advanced spinal synaptic facilitation and low VDT a loss of Aβ-fiber function. Thus from a physiological point of view, the postulated relationship between the probability to respond to acupuncture and these sensory parameters is most likely not linear. This was accounted for by categorizing TS and VDT values in the statistical analysis (see section Data Analysis).

### Temporal Summation of Pain

As a measure for TS, the pin-prick evoked wind-up ratio (WUR) was applied. The WUR represents the quotient of the pain intensity evoked by 10 pin-prick stimuli and the pain intensity evoked by one single pin-prick stimulus. For all measurements a pin-prick of 256 mN (*MRC Systems GmbH-Medizintechnische Systeme, Heidelberg, Germany*) was used. Patients were asked to rate the pin-prick evoked pain intensity on the VRS (0–100). First, the researcher applied a single pin-prick stimulus followed by a series of 10 pin-prick stimuli applied with a frequency of 1 Hz within an area of 1 cm^2^. WUR assessments were repeated three times per measurement site at intervals of at least 15 s. The WUR was calculated by division of the arithmetic mean of the three VRS ratings of the 10 stimuli by the arithmetic mean of the three VRS ratings of the single stimuli. This is indicated by the denotation [VRS-ratio], since the wind-up ratio is a dimensionless quantity.

#### Vibration Detection Threshold

The VDT was evaluated by the Rydel-Seiffer tuning fork (*Aesculap AG, B.Braun Melsungen AG, Melsungen, Germany*) which allows the application of vibration stimuli of 64 Hz. The continuously decreasing vibration amplitude can be read from a gauged scale ranging from 0 to 8 with an accuracy of 0.5 points. Patients were asked to notify the examiner as soon as their sensation of vibration stopped, and the corresponding amplitude was recorded. The final VDT value also represents the arithmetic mean of three measurements.

### Assessment of Patient Characteristics

Sociodemographic and pain diagnostic data were extracted from the patients' medical records. These data included age in years, sex, right-handedness, pain relevant, and psychological diagnoses according to ICD-10 coding, stage of chronicity according to the Mainz-Pain Staging System (MPSS, 1–3), previous surgery (yes/no) and use of analgesics (yes /no; if yes, class of drugs was recorded). Pain duration in months, the most painful body site as well as the average and maximum pain intensity (VRS, 0-100) were assessed during the first study visit on the first treatment day.

### Sample Size Estimation

Sample size estimation was performed on the basis of reference data for the WUR provided by the DFNS which suppose a log-normal data-distribution (Rolke et al., 2006). Given a target difference of 0.4 of WUR raw-values (0.15 in log-values), a standard deviation (SD) of 0.234 in log-values, an α-error of 5% and a power of 80%, the total sample size was estimated as 78 patients. In addition, a correction for an unequal responder/non-responder ratio of 33 to 66% and a drop-out rate of 15% resulted in a final sample size of 101 patients (calculations according to Altman, 1991).

### Data Analysis

Data analysis was exploratory and performed in IBM SPSS Statistics for Windows (Version 22.0. Armonk, NY: IBM Corp) as well as in R version 3.5.2 (R Development Core Team, 2008). Categorical study variables are described as absolute and relative frequencies. Assumption of normal distribution of metric study variables was estimated by Shapiro-Wilk test. Besides age all metric study variables (pain intensity measures, pain duration, WUR and VDT) were not normally nor log-normally distributed. Descriptive statistics are therefore given as median with interquartile range (IQR) for the remaining metric variables.

A responder analysis was carried out to evaluate whether the predictors were associated with clinically relevant treatment response. As described above, percent changes in VRS sores were categorized (≥30 and ≥50%) to represent responders and non-responders. As recommended for analyses of pain data (Farrar et al., 2006), cumulative proportion of responder analysis graphs are used to illustrate that cut-off selection did not bias results. That this constitutes the best strategy to present our data is based on two main considerations. First, neither raw post-treatment scores nor raw change scores nor percent change scores provide an unbiased estimate of treatment effects (Vickers, 2001). Second, regression analyses with adjustment for baseline scores is not recommended in observational studies, in particular if covariates are likely to be related to baseline scores (Cribbie and Jamieson, 2000; Cole et al., 2010).

Logistic regression was used to assess confounder adjusted associations between the dichotomous outcome parameters representing acupuncture effects (dependent variables) and the VDT as well as the WUR at the control site and at the pain site (independent variables). Based on the neurophysiological considerations introduced above, VDT and WUR data were categorized before application as independent variables in the logistic regression models. We pragmatically chose the 33%- percentile for the VDT and the 66%-percentile for the WUR as cut-off values in order to identify patients with the highest WUR and lowest VDT. These cut-off values were verified by visual inspection of scatter plots of predictors against percent change in pain intensity (**Figure 2**) and ROC-analyses.

Assessed confounders included age, gender, pain duration, pain related surgery before participation in the multimodal pain treatment and intake of analgesics. First, the crude associations of acupuncture effects and patient characteristics were assessed in an exploratory analysis. Subgroups formed according to the different dichotomous outcome variables were compared for metric variables by Mann-Whitney-U test and for categorical variables by Fisher test. Subsequently, final logistic regression models were identified by automated covariate selection using the R-function *bestglm* (McLeod and Changjiang, 2018). Model fit was evaluated by Omnibus test and goodness of fit by Hosmer-Lemeshow test. Odds-ratios (OR) with 95% confidence intervals (95% CI), the Nagelkerke's pseudo *R*^2^ (*R*^2^) as well as correctly specified cases (sensitivity & specificity) were used to describe the predictive value of the VDT and the WUR at the control site and at the pain site. Collinearity was assessed by the variation inflation factor (VIF). Effect modification was assessed by introduction of interaction terms in the final models. For additional graphical illustration (e.g., cumulative response curves), identified metric confounders were categorized with cut-off values identified by the receiver operating characteristic (ROC-) analysis.

## Results

### Patient Characteristics

**Figure 1:**
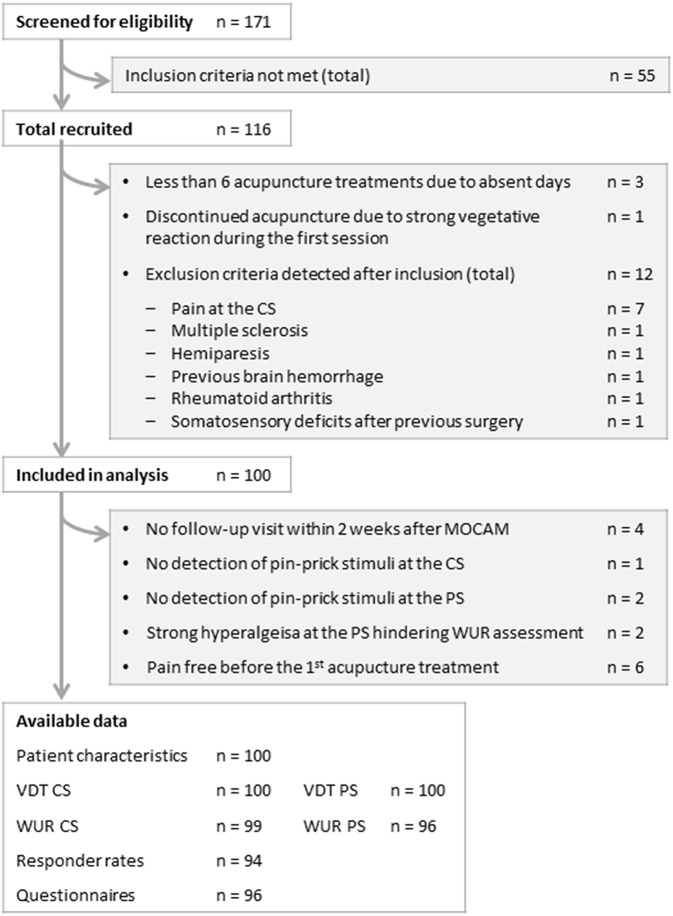
Study flow-chart. MOCAM, Munich Outpatient Program in Complementary and Alternative Medicine; VDT, vibration detection threshold; WUR, wind-up ratio; CS, control site; PS, pain site.

Of 171 screened patients 116 met the inclusion criteria and gave written informed consent. Of these 16 were excluded from the analysis. A late detection of exclusion criteria accounted for exclusion in 12 cases. Three patients had received less than six acupuncture treatments during the multimodal treatment program for organizational reasons, and one patient discontinued the acupuncture treatment after experiencing a strong vegetative reaction during the first session. Four patients did not attend the follow-up visits for personal reasons (Figure 1).

Patient characteristics of the 100 patients available for analysis are displayed in Table 1. Overall, patients were middle-aged, mainly females, right-handed, with a long history of chronic pain and relevant pain intensity. Most patients (n = 77) were diagnosed with persistent pain with somatic and psychological factors (F45.41). The second most frequent diagnosis (n = 64) was myofascial pain (M79.1-) followed by dorsopathies (M40–54, *n* = 42). Migraine or other headache syndromes (G43.- or G44.-) were present in 21, arthropathies (M00–25) in 18 and disorder of the trigeminal nerve (G50.-) in 12 patients. The most frequent mental and behavioral diagnoses were mood or affective disorder (F30–39) present in 34 patients. The most frequently reported area of the main pain problem was the lower back (25%) followed by the neck and cervical-spine area (22%), the shoulder-arm region (10%), the head (9%) and the area of the thoracic spine (8%).

**Table 1:** Patient characteristics (*n* = 100).

**Socidemographic and clinical characteristics**			**Diagnoses established during an interdisciplinary pain assessment**	**ICD-10**	***n***
Age [a], mdn (IQR)		50 (43–56)	Persistent pain with somatic and mental factors	F45.41	77
Female/male, *n*		83/17	Migraine and other headache syndromes	G43.-/ G44.-	21
Right handed, *n*		98	Disorders of trigeminal nerve	G50.-	4
Pain duration [m], mdn (IQR)		66 (22–187)	Mononeuropathies of upper limb	G56.-	2
Pain intensity VRS (0–100) mdn (IQR)	Maximum	80 (61–90)	Tinnitus	H93.1	1
	Average	50 (40–60)	Disorders of circulatory system	I73.9/ I80.28	2
	Current	45 (30–60)	Temporomandibular joint disorders	K07.6	5
Chronicity	1	7	Arthropathies	M00-25	18
MPSS, *n*	2	38	Polymyalgia rheumatica	M35.3	1
	3	55	Deforming dorsopathies	M40–43	3
Previous surgery because of chronic pain, *n*		29	Spondylopathies	M45–49	2
Main pain area, *n*	Lower back	25	Other dorsopathies	M50-54	37
	Neck/cervical-spine area	22	Shoulder lesions	M75.-	7
	Thoracic spine-area	8	Enthesopathies of lower limb, excluding foot	M76.-	5
	Headache	9	Other enthesopathies	M77.-	5
	Jaw	7	Myalgia, myofascial pain syndromes	M79.1-	64
	Shoulder/arm	10	Pain in limb	M79.6-	1
	Hip	5	Fibromyalgia	M79.70	8
	Knee, lower extremity	6	Osteoporosis without pathological fracture	M81.-	2
	Foot	6	Osteomyelitis	M86.-	1
	Abdomen	2	Other biomechanical lesions	M99.8-	7
Pain medication, *n*	Non-opioid analgesics (NSAID/	68	Other congenital deformities of feet	Q66.8	1
	Pyrazolone/Paracetamole)		Symptoms and signs abdomen/urinary system	R10.1/ R39.8	2
	Weak opioids	12	Pain, unspecified	R52.9	1
	Antidepressants	23	Fracture of thoracic vertebra	S22.0	1
	Anticonvulsants	9	Presence of other functional implants	Z96.-	2
	Triptans	6	Mental and behavioral disorders due to	F10 - F19	7
	Muscle relaxants	2	psychoactive substance use		
	Disease-modifying	1	Mood [affective] disorders	F30 - F39	34
	antirheumatic drugs		Neurotic, stress-related and somatoform disorders	F40 - F48	22
	Phytopharmaceuticals	4	Behavioral syndromes associated with	F50 - F59	12
	No analgesics	15	physiological disturbances and physical factors		

*n, absolute frequency corresponding to relative frequencies since the total number of patients was 100; a, years; m, months; mdn, median; IQR, interquartile range; VRS, verbal rating scale; MPSS, Mainz Pain Staging System; ICD-10, 10th revision of the International Statistical Classification of Diseases and Related Health Problems*.

### Descriptive Analysis of Acupuncture Effects, TS and the VDT

Pain intensity as evaluated by the VRS (0–100) was significantly reduced after the first acupuncture treatment when compared to baseline (median (IQR) 30 (10–50) vs 45 (30–60), *p* < 0.001).

Six patients had no pain on the first day of MOCAM and were therefore excluded from responder rate calculations. Of those suffering from pain on the first day of the multimodal pain treatment, 38 (40.4%) experienced a pain reduction of at least 30% (Resp_30_) and 25 (26.6%) of at least 50% (Resp_50_) after the first acupuncture treatment as evaluated by the VRS (0–100).

Thirty seven patients (38.5%) reported that they had experienced an immediate pain relief through acupuncture in the questionnaire filled in after the end of the 4 weeks of treatment. Twenty-four patients (25%) affirmed that acupuncture had reduced their pain immediately by over 50%. Subjective rating of the contribution of acupuncture to the overall pain relief over the whole 4-week treatment period was positive in 52 cases (54.7%). Seventeen patients (17.9%) perceived acupuncture to be responsible for an overall pain relief of over 50%. Seventy-five patients (80.6%) experienced a beneficial effect of acupuncture on general well-being.

TS was similar at the control site and at the pain site with a median WUR (IQR) of 1.9 (1.3–3.0) and 2.0 (1.5–3.1), respectively. Insensitivity to pin-prick stimuli (VRS rating of 1 stimulus with 0) leading to missing values of the WUR were observed in one patient at the control site and in two patients at the pain site. In two patients TS assessments at the pain site could not be performed due to strong hyperalgesia.

Median VDT at the control site was 8.0 with an IQR of 7.0–8.0 indicating a ceiling effect (accumulation of scores at the upper limit of the 8-point scale). At the pain site median VDT was 5.4 (IQR 4.0–6.6).

### Crude Association of TS and the VDT With Acupuncture Effects

Comparisons of TS and the VDT at the control site and at the pain site were performed between patient subgroups formed according to the different dichotomous outcome variables: reduction of the pain intensity as evaluated by the VRS after the first acupuncture treatment over 30% (Resp_30_) or 50% (Resp_50_), subjective perception of acupuncture effects as assessed by the questionnaire—immediate pain relief, immediate pain relief of over 50%, contribution to overall pain relief after 4 weeks of treatment, overall pain relief over 50% and contribution to overall well-being (Table 2). TS at the control site was significantly higher in patients who reported an immediate pain relief through acupuncture in the questionnaire than in those who did not experience an immediate analgesic acupuncture effect (median WUR (IQR) 2.4 (1.5–3–6) vs. 1.6 (1.3–2.8); *p* = 0.050). Patients who had reported an immediate pain relief of over 50% were even more demarked form the remaining patients in terms of a higher TS at the control site (median WUR (IQR) 2.5 (1.6–4.4) vs. 1.6 (1.3–2.9); *p* = 0.029). In addition, there was a trend toward higher TS at the pain site in patients with an immediate reduction in their pain intensity after the first acupuncture treatment of more than 50% (Resp_50_) as evaluated by the VRS (*p* = 0.085). Conversely, patients with and without acupuncture effects differed neither with regard to the VDT at the control site nor with regard to the VDT at the pain site (*p* > 0.05).

**Table 2:** TS and VDT in patients with and without acupuncture effect.

		**Resp**_****30****_			**Resp**_****50****_		
		**Yes**	**No**	***p*-value**	**Yes**	**No**	***p*-value**
VDT CS	*n*	38	56		25	69	
(x/8)	mdn [IQR]	7.8 [7.5–8.0]	8 [7.5–8.0]	0.346	7.8 [7.5–8.0]	8.0 (7.5–8.0)	0.61
VDT PS	*n*	38	56		25	69	
(x/8)	mdn [IQR]	5.9 [4.3–6.7]	5.3 [3.9–6.3]	0.135	5.7 [4.3–6.7]	5.3 (4.0–6.4)	0.353
WUR CS	n	38	55		25	68	
(VRS-ratio)	mdn [IQR]	2.4 [1.4–3.4]	1.7 [1.3–2.8]	0.189	2.3 [1.4–3.4]	1.7 (1.3–3.0)	0.553
WUR PS	*n*	37	53		25	65	
(VRS-ratio)	mdn [IQR]	2.0 [1.6–4.0]	1.8 [1.5–3.0]	0.124	2.0 [1.6–3.2]	1.9 (1.5–3.1)	0.479
		**Subjective immediate pain relief**			**Subjective immediate pain relief** **>50%**		
		**Yes**	**No**	**p-value**	**Yes**	**No**	***p-*****value**
VDT CS	n	37	59		24	72	
(x/8)	mdn [IQR]	8.0 [7.6–8.0]	8.0 [7.5–8.0]	0.452	8.0 [7.7–8.0]	8.0 (7.5–8.0)	0.345
VDT PS	*n*	37	59		24	72	
(x/8)	mdn [IQR]	5.7 [4.6–6.7]	5.3 [4.0–6.5]	0.265	5.7 [4.4–7.0]	5.3 (4.2–6.5)	0.367
WUR CS	*n*	36	59		23	72	
(VRS-ratio)	mdn [IQR]	2.4 [1.5–3.6]	1.6 [1.3–2.8]	0.050^*^	2.5 [1.6–4.4]	1.6 (1.3–2.9)	0.029^*^
WUR PS	*n*	36	56		23	69	
(VRS-ratio)	mdn [IQR]	2.3 [1.6–3.3]	1.9 [1.5–2.6]	0.13	2.8 [1.6–3.4]	1.9 (1.5–2.7)	0.085
		**Subjective overall pain relief**			**Subjective overall pain relief** **>50%**		
		**Yes**	**No**	***p*****-value**	**Yes**	**No**	***p*****-value**
VDT CS	*n*	52	43		17	78	
(x/8)	mdn [IQR]	8.0 [7.5–8.0]	8.0 [7.7–8.0]	0.373	8.0 [7.4–8.0]	8.0 (7.5–8.0)	0.953
VDT PS	*n*	52	43		17	78	
(x/8)	mdn [IQR]	5.5 [4.0–6.6]	5.7 [4.5–6.7]	0.381	4.7 [4.0–6.3]	5.7 (4.3–6.7)	0.319
WUR CS	*n*	51	43		16	78	
(VRS-ratio)	mdn [IQR]	1.7 [1.3–3.0]	2.0 [1.3–2.8]	0.381	1.7 [1.6–3.0]	1.9 (1.3–3.0)	0.856
WUR PS	*n*	50	42		17	75	
(VRS-ratio)	mdn [IQR]	2.0 [1.5–2.8]	2.0 [1.4–3.2]	0.931	2.0 [1.5–2.7]	2.0 (1.5–3.1)	0.629
		**Contribution to general well-being**					
		**Yes**	**No**	***p*****-value**			
VDT CS	*n*	75	18				
(x/8)	mdn [IQR]	8.0 [7.5–8.0]	8.0 [7.6–8.0]	0.901			
VDT PS	*n*	75	18				
(x/8)	mdn [IQR]	5.7 [4.2–6.7]	5.3 [4.0–6.6]	0.942			
WUR CS	n	74	18				
(VRS - ratio)	mdn [IQR]	1.7 [1.3–3.0]	2.2 [1.4–3.1]	0.598			
WUR PS	*n*	71	18				
(VRS-ratio)	mdn [IQR]	2.0 [1.5–3.0]	2.3 [1.5–3.4]	0.314			

Resp_30/50_, Acupuncture responders defined according to a reduction in pain intensity after the first acupuncture treatment of at least 30 and 50%, respectively; VDT, vibration detection threshold; x/8, score out of eight on the Rydel-Seiffer tuning fork; WUR, wind-up ratio; VRS-ratio, ratio of the pain intensities evoked by one and by ten pin-prick stimuli of 256 mN as evaluated by the verbal rating scale (0–100); CS, control site; PS, pain site; n, case number; mdn, median; IQR, interquartile range;

* *statistically significant at an α-error of 5% according to Mann-Whitney-U test*.

**Figure 2:**
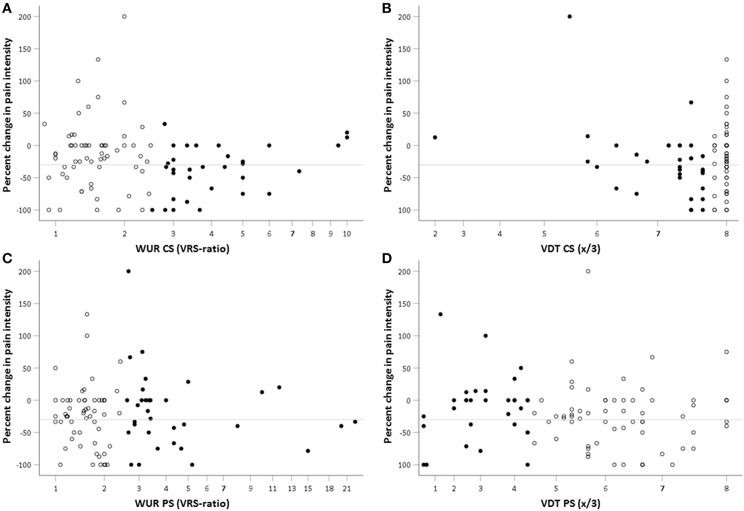
Scatterplot of percent change in pain intensity after the first acupuncture treatment against WUR and VDT at the PS and CS. Percent change in pain intensity as evaluated by the verbal rating scale (VRS) was calculated as (VRSpost – VRSpre)/VRSpre ^*^100. Accordingly, negative values represent a pain relief. The reference line indicates a 30% reduction in pain intensity as the cut-off for clinically relevant pain relief. WUR, wind-up ratio [ratio of the pain intensities evoked by one and by 10 pin-prick stimuli of 256 mN as evaluated by the verbal rating scale (0–100)]; VDT, vibration detection threshold; CS, control site; PS, pain site; filled points, **(A,B)** subjects with high TS (WUR > 2.5 VRS-ratio) as identified by the 66%-percentile **(C)** low VDT at the CS ( ≤ 7.7/8) **(D)** low VDT at the PS ( ≤ 4.3/8) as identified by the 33%- percentile; empty points, subjects with low TS or high VDT, respectively.

Plotting percent change in pain intensity against the predictor variables showed a non-linear relationship between the WUR and the immediate reduction in pain intensity after acupuncture. The density of data points representing no or only poor acupuncture response decreases in the value range between 2 and 3 of the WUR at the control site (Figure 2A). A similar but less pronounced association was seen for TS at the pain site (Figure 2B). In contrast, visual inspection does not suggest an association between percent change in pain intensity and the VDT at the pain site or at the control site (Figures 2C,D).

Comparisons of frequencies of low VDT and high TS between patient subgroups with and without acupuncture effects (Table 3) support observations from Figure 2. High TS (WUR > 2.5) at the control site was more frequent in patients with a reduction in pain intensity of at least 30% (Resp_30_) as evaluated by the VRS than in patients not responding to the first treatment (OR 2.6 [1.1–6.4]; *p* = 0 0.045). The proportion of patients with high TS at the control site was also larger among those who subjectively rated the immediate pain relief through acupuncture as positive (OR 2.6 [1.1–6.3]; *p* = 0.043) or even over 50% (OR 2.8 [1.1–7.5]; *p* = 0.043). High TS at the pain site was merely associated with the report of an immediate pain relief through acupuncture of over 50% in the questionnaire (OR 3.4 [1.3–9.1]; *p* = 0.021). In addition, a ROC-analysis confirmed the WUR cut-off of 2.5 for all outcomes. Both a low VDT at the control site ( ≤ 7.7) and a low VDT at the pain site ( ≤ 4.3) were equally frequent in patients with and without acupuncture effects.

**Table 3:** Crude association between acupuncture effects and high TS and low VDT.

		**Resp**_****30****_				**Resp**_****50****_			
		**Yes**	**No**	**OR [95% CI]**	***p-*value**	**Yes**	**No**	**OR [95% CI]**	***p*-value**
VDT control site (x/8)	>7.7	21	39	ref		15	45	ref	
	≤ 7.7	17	17	1.9 [0.8–4.4]	0.191	10	24	1.3 [0.5–3.2]	0.637
VDT pain site (x/8)	>4.3	28	35	ref		18	45	ref	
	≤ 4.3	10	21	0.6 [0.2–1.5]	0.275	7	24	0.7 [0.3–2.0]	0.625
WUR control site (VRS-ratio)	≤ 2.5	20	41	ref		14	47	ref	
	>2.5	18	14	2.6 [1.1–6.4]	0.045^*^	11	21	1.8 [0.7–4.5]	0.325
WUR pain site (VRS-ratio)	≤ 2.5	21	36	ref		16	41	ref	
	>2.5	16	17	1.6 [0.7–3.8]	0.374	9	24	1.0 [0.4–2.5]	1.000
		**Subjective immediate pain relief**				**Subjective immediate pain relief** **>50%**			
		**Yes**	**No**	**OR [95% CI]**	***p*****-value**	**Yes**	**No**	**OR [95% CI]**	***p*****-value**
VDT control site (x/8)	>7.7	24	38	ref		17	45	ref	
	≤ 7.7	13	21	1.0 [0.4–2.3]	1.000	7	27	0.7 [0.3–1.9]	0.623
VDT pain site (x/8)	>4.3	29	37	ref		18	48	ref	
	≤ 4.3	8	22	0.5 [0.2–1.2]	0.120	6	24	0.7 [0.2–1.9]	0.612
WUR control site (VRS-ratio)	≤ 2.5	19	44	ref		11	52	ref	
	>2.5	17	15	2.6 [1.1–6.3]	0.043^*^	12	20	2.8 [1.1–7.5]	0.043^*^
WUR pain site (VRS-ratio)	≤ 2.5	19	41	ref		10	50	ref	
	>2.5	17	15	2.4 [1.0–5.9]	0.072	13	19	3.4 [1.3–9.1]	0.021^*^
		**Subjective overall pain relief**				**Subjective overall pain relief** **>50%**			
		**Yes**	**No**	**OR [95% CI]**	***p*****-value**	**Yes**	**No**	**OR [95% CI]**	***p*****-value**
VDT control site (x/8)	>7.7	32	30	ref		11	51	ref	
	≤ 7.7	20	13	1.4 [0.6–3.4]	0.517	6	27	1.0 [0.3–3.1]	1.000
VDT pain site (x/8)	>4.3	32	33	ref		9	56	ref	
	≤ 4.3	20	10	2.1 [0.8–5.1]	0.127	8	22	2.3 [0.8–6.6]	0.155
WUR control site (VRS-ratio)	≤ 2.5	32	30	ref		10	52	ref	
	>2.5	19	13	1.4 [0.6–3.2]	0.518	6	26	1.2 [0.4–3.7]	0.777
WUR pain site (VRS-ratio)	≤ 2.5	35	24	ref		12	48		
	>2.5	15	17	0.6 [0.3–1.4]	0.380	5	27	0.7 [0.2–2.3]	0.780
		**Contribution to general well-being**							
		**Yes**	**No**	**OR [95% CI]**	***p*****-value**				
VDT control site (x/8)	>7.7	49	12	ref					
	≤ 7.7	26	6	1.1 [0.4–3.2]	1.000				
VDT pain site (x/8)	>4.3	52	13	ref					
	≤ 4.3	23	5	1.2 [0.4–3.6]	1.000				
WUR control site (VRS-ratio)	≤ 2.5	48	13	ref					
	>2.5	26	5	1.4 [0.5–4.4]	0.782				
WUR pain site (VRS-ratio)	≤ 2.5	47	11	ref					
	>2.5	24	7	0.8 [0.3–2.3]	0.783				

Resp_30/50_, Acupuncture responders defined according to a reduction in pain intensity after the first acupuncture treatment of at least 30 or 50%, respectively; VDT, vibration detection threshold; x/8, score out of eight on the Rydel-Seiffer tuning fork; WUR, wind-up ratio; VRS-ratio, ratio of the pain intensities evoked by one and by ten pin-prick stimuli of 256 mN as evaluated by the verbal rating scale (0–100); CS, control site; PS, pain site; OR, odds ratio; 95% CI, 95% confidence interval;

* *statistically significant at an α-level of 5% according to Fisher test*.

### Associations Between Acupuncture Effects and Patient Characteristics

Acupuncture effects were similarly distributed between men and women. There was only a trend toward a more frequent perception of an immediate pain relief of over 50% through acupuncture in females (OR 6.6 [0.8–52.5]; *p* = 0.062). All 24 patients who had reported an immediate pain relief through acupuncture of over 50% in the questionnaire took analgesics, while 20.8% (15 out of 72) of patients who experienced less or no immediate pain relief after one of the acupuncture sessions were not on pain medication (*p* = 0.019). Patients of chronicity stage 2 and 3 as defined by the MPSS rated the contribution of acupuncture to their general wellbeing more frequently as positive than patients of chronicity stage 1 (OR 7.7 [1.3–45.4]; *p* = 0.031 and OR 6.4 [1.2–33.5]; *p* = 0.036, respectively). Furthermore, older age was associated with immediate analgesic response to acupuncture. Patients with immediate, clinically relevant reductions in VRS pain scores were older than patients without such response (Resp_30_: mean (SD) 51.7 (9.4) vs. 46.4 (10.8) years, *p* = 0.005; Resp_50_: (52.8 (9.6) vs. 47.0 (10.6) years, *p* = 0.006). The subjective experience of an immediate pain relief through acupuncture of over 50% was also associated with older age (53.3 (8.4) vs. 46.3 (11.3) years; *p* = 0.010). A similar age trend was also observed between patient subgroups formed according to whether an immediate pain relief was experienced at all (50.8 (11.2). vs 46.3 (10.7) years; *p* = 0.063) (Table 4).

**Table 4:** Crude associations between acupuncture effects and patient characteristics.

		**Resp**_****30****_				**Resp**_****50****_			
		**Yes**	**No**	**OR [95% CI]**	***p*-value**	**Yes**	**No**	**OR [95% CI]**	***p*-value**
Gender	Male	3	11	ref		1	13	ref	
	Female	35	45	2.9 [0.7–11.0]	0.147	24	56	5.6 [0.7–45.0]	0.103
Chronicity MPSS	1	0	5	ref		0	5	ref	
	2	14	20	-	0.139	7	27	-	0.563
	3	24	31	-	0.077	18	37	-	0.310
Surgery	No	28	39	ref		17	50	ref	
	Yes	10	17	0.8 [0.3–2.1]	0.817	8	19	1.2 [0.5–3.3]	0.797
Use of analgesics	No	2	10	ref		1	11		
	Yes	36	46	3.9 [0.8–19.0]	0.114	24	58	4.6 [0.6–37.2]	0.172
Pain duration [m]	mdn (IQR)	48 (16–166)	116 (26–219)		0,119	53 (14–145)	82 (24–218)		0,176
Age [a]	mdn (IQR)	53 (46–59)	49 (42–51)		0.005^*^	55 (48–61)	49 (42–52)		0.006^*^
		**Subjective immediate pain relief**				**Subjective immediate pain relief** **>50%**			
		**Yes**	**No**	**OR [95% CI]**	***p*****-value**	**Yes**	**No**	**OR [95% CI]**	***p*****-value**
Gender	Male	5	12	ref		1	16	ref	
	Female	32	47	1.6 [0.5–5.1]	0.584	23	56	6.6 [0.8–52.5]	0.062
Chronicity MPSS	1	2	5	ref		1	6	ref	
	2	12	23	1.3 [0.2–7.8]	1.000	10	25	2.4 [0.3–22.6]	0.654
	3	23	31	1.9 [0.3–10.4]	0.690	13	41	1.9 [0.2–17.3]	1.000
Surgery	No	25	42	ref		16	51	ref	
	Yes	12	17	1.2 [0.5–2.9]	0.820	8	21	1.2 [0.5–3.3]	0.798
Use of analgesics	No	4	11	ref		0	15	ref	
	Yes	33	48	1.9 [0.6–6.5]	0.393	24	57	-	0.019^*^
Pain duration [m]	mdn (IQR)	85 (21–217)	49 (21–166)		0.372	65 (17–219)	66 (21–182)		0.889
Age [a]	mdn (IQR)	51 (46–59)	50 (42–52)		0.063	55 (47–60)	50 (41–52)		0.010^*^
		**Subjective overall pain relief**				**Subjective overall pain relief** **>50%**			
		**Yes**	**No**	**OR [95% CI]**	***p*****-value**	**Yes**	**No**	**OR [95% CI]**	***p-*****value**
Gender	Male	10	7	ref		2	15	ref	
	Female	42	36	0.8 [0.3–2.4]	0.792	15	63	1.8 [0.4–8.7]	0.729
Chronicity MPSS	1	4	3	ref		2	5	ref	
	2	15	19	0.6 [0.1–3.1]	0.685	3	31	0.2 [0.0–1.8]	0.196
	3	33	21	1.2 [0.2–5.8]	1.000	12	42	0.7 [0.1–4.2]	0.655
Surgery	No	37	29	ref		13	53	ref	
	Yes	15	14	0.8 [0.3–2.0]	0.823	4	25	0.7 [0.2–2.2]	0.573
Use of analgesics	No	8	7	ref		3	12	ref	
	Yes	44	36	1.1 [0.4–3.2]	1.000	14	66	0.8 [0.2–3.4]	0.728
Pain duration [m]	mdn (IQR)	87 (18–229)	49 (21–166)		0.267	123 (15–272)	63 (21–173)		0.344
Age [a]	mdn (IQR)	50 (43–55)	51 (43–57)		0.837	49 (45–57)	51 (43–56)		0.973
		**Contribution to general well-being**							
		**Yes**	**No**	**OR [95% CI]**	***p*****-value**				
Gender	Male	12	5	ref					
	Female	63	13	2.0 [0.6–6.7]	0.308				
Chronicity MPSS	1	3	4	ref					
	2	29	5	7.7 [1.3–45.5]	0.031^*^				
	3	43	9	6.4 [1.2–33.5]	0.036^*^				
Surgery	No	53	12	ref					
	Yes	22	6	0.8 [0.3–2.5]	0.778				
Use of analgesics	No	10	5	ref					
	Yes	65	13	2.5 [0.7–8.5]	0.158				
Pain duration [m]	mdn (IQR)	66 (21–182)	19 (59–202)		0.911				
Age [a]	mdn (IQR)	50 (41–52)	49 (34–54)		0.549				

Resp_30/50_, Acupuncture responders defined according to a reduction in pain intensity after the first acupuncture treatment of at least 30 or 50%, respectively; MPSS, Mainz Pain Staging System; m, months; a, years; ref, reference category; mdn, median; IQR, interquartile range; OR, odds ratio; 95% CI, 95% confidence interval;

* *statistically significant at an α-level of 5% according to Fisher test for dichotomous variables and according to Mann-Whitney-U test for continuous variables*.

### Adjusted Analysis of the Association Between TS and the VDT With Acupuncture Effects

Logistic regression confirmed the positive association of high TS at the control site as well as at the pain site with an immediate reduction in pain after acupuncture and identified age as a relevant confounder (Model 1–4; Table 5). Age-adjusted ORs were larger than crude ORs (Table 4). All models showed a good model fit (Omnibus test *p* < 0.01 and H-L test > 0.07). There was no collinearity between explanatory variables (VIF <1.1). Explained variance according to Nagelkerkes *R*^2^ was between 18 and 25%. As identified by automated covariate selection, age was the only additional significant explanatory variable. Effect modification by any patient characteristic was not observed. Specificity of prediction (80–94%) was usually larger than sensitivity (13–55%).

**Table 5:** Logistic regression models of age-adjusted associations between immediate analgesic acupuncture effects and high TS.

		**Model 1**	**Model 2**	**Model 3**			**Model 4**
**Dependent variable**		**Resp_**30**_**	**Subjective immediate pain relief**	**Subjective immediate pain relief >50%**	**Dependent variable**		**Subjective immediate pain relief >50%**
**Model fit criteria**					**Model fit criteria**		
WUR control site >2.5 (VRS-ratio)	OR	4.3	3.8	5.5	WUR pain site >2.5 (VRS-ratio)	OR [95%-KI]	3.2
	[95%-KI]	[1.6–11.8]	[1.4–10.2]	[1.7–17.1]			[1.2–8.9]
	*p-value*	0.005^*^	0.007^*^	0.003^*^		*p-value*	0.024^*^
Age [a]	OR	1.08	1.06	1.11	Age [a]	OR	1.07
	[95%-KI]	[1.0–1.1]	[1.0–1.1]	[1.0–1.2]		[95%-KI]	[1.0–1.1]
	*p-value*	0.004^*^	0.014^*^	0.002^*^		*p-value*	0.026^*^
Constant	OR	0.01	0.02	0.00	Constant	OR	0.01
	*p-value*	0.001^*^	0.003^*^	<0.001^*^		*p-value*	0.002^*^
Omnibus test	*p-value*	0.001^*^	0.003^*^	<0.001^*^	Omnibus test	*p-value*	0.002^*^
H-L test	*p-value*	0.407	0.311	0.169	H-L test	*p-value*	0.076
Nagelkerkes R^2^		0.199	0.157	0.251	Nagelkerkes R^2^		0.183
VIF		1.056	1.056	1.056	VIF		1.034
Correctly classified cases [%]	Yes No Total	55.3 80.0 69.9	36.1 86.4 67.4	26.1 94.4 77.9	Correctly classified cases [%]	Yes No Total	13.0 94.2 73.9

Resp_30_, Acupuncture responders defined according to a reduction in pain intensity after the first acupuncture treatment of at least 30%; WUR, wind-up ratio; VRS-ratio, ratio of the pain intensities evoked by one and by ten pin-prick stimuli of 256 mN as evaluated by the verbal rating scale (0–100); CS, control site; PS, pain site; a, years; OR, odds ratio; 95% CI, 95% confidence interval; H-L test, Hosmer-Lemeshow test; VIF, variation inflation factor;

* *statistically significant at an α-level of 5%*.

Model 1 describes the association between a high TS at the control site and the outcome Resp_30_. It indicates that patients with a high TS (WUR > 2.5) at the control site had a 4.0 (95% CI [1.6–11.8]; *p* = 0.005) times higher chance that their pain intensity was reduced by at least 30% after the first acupuncture treatment. With each year of age the chance to belong to the Resp_30_-subgroup increased by 8% (95% CI [2–13%]; *p* = 0.004). The model correctly classified 55% of responders and 80% of non-responders.

**Figure 3:**
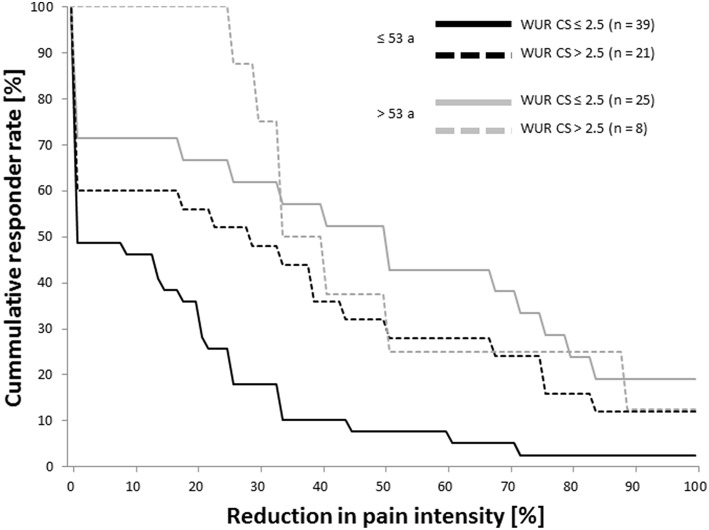
Cumulative response functions in patients with high and low TS at the control site by age-group. Reduction in pain intensity was evaluated by the verbal rating scale (0–100) before and after the first acupuncture treatment. The age cut-off of 53 years was estimated by a receiver operating characteristic analysis. TS, temporal summation of pain; WUR, wind-up ratio [ratio of the pain intensities evoked by one and by ten pin-prick stimuli of 256 mN as evaluated by the verbal rating scale (0–100)]; CS, control site; a, years.

There was no significant association between a reduction in pain intensity after the first acupuncture treatment of over 50% (Resp_50_) and a high TS (WUR > 2.5). However, when subgrouping patients according to an age cut-off identified by a ROC-analysis (53 years), the cumulative response function (Figure 3) shows a trend toward higher frequencies of responders among those with a WUR over 2.5 irrespective of the responder definition. Responder rates in the subgroup of patients over 53 years of age displaying a high WUR are only based on eight cases.

Model 2 indicates that the subjective experience of an immediate pain relief through acupuncture was reported with a 3.8 (95% CI [1.4–10.2%]; *p* = 0.007) times higher odds in patients with a high TS (WUR > 2.5) at the control site. With every year of age the likelihood of such positive appraisal of the immediate analgesic effect of acupuncture increased by 6% (95% CI [1–11%]; *p* = 0.014). Specificity was slightly higher (86%) and sensitivity lower (36%) than in model 2.

The association between a subjective immediate pain relief of over 50% after one of the acupuncture sessions with a high TS at the control site is described by model 3. Patients with a WUR of over 2.5 at the control site were 5.5 (95% CI [1.7–17.1]; *p* = 0.003) times more likely to report such strong immediate analgesic response. The influence of age was larger when compared to the other final models (OR 1.11 [1.04–1.189]; *p* < 0.001). With 78% the percentage of correctly classified cases was also high when compared to the other models. Specificity was 94%, while sensitivity was lower than in the other models (26%).

According to model 4, a high TS (WUR > 2.5) at the pain site increased the chance to experience an immediate pain relief of over 50% after one of the acupuncture treatments by 3.2 (95% CI [1.2–8.9]; *p* = 0.024) times. The odds was further increased by 7% (95% CI [1–14%]; *p* = 0.026) with every year of age. As in model 3, specificity was as high as 94% while the sensitivity of 13% was the lowest among all models.

## Discussion

To our knowledge, this is the first study evaluating temporal summation of pain (TS) and the vibration detection threshold (VDT) as predictors for acupuncture effects. The results support the hypothesis that high TS might predict response to acupuncture in chronic pain patients with regard to immediate effects. The presumed association of vibration detection with acupuncture effects was not ascertained.

### Association of High Temporal Summation of Pain With Acupuncture Effects

A WUR over 2.5 indicating elevated TS at a pain-free control site and at the most painful body site increased the likelihood to experience immediate analgesic effects through acupuncture by three to five times. This suggests that in chronic pain patients in whom spinal synaptic transmission is facilitated acupuncture may be particularly effective for immediate pain relief. This appears plausible considering that immediate analgesic acupuncture effects are mainly mediated by bottom-up and top-down mechanisms which reduce spinal transmission (Zhao, 2008; Zhang et al., 2014). These include the activation of the descending inhibitory pain control, segmental inhibition, a reduction of spinal pronociceptive peptides and local modulation of primary afferents mediated by purinergic signaling.

Increased TS has been shown to be associated with the efficacy of drugs i.e., Ketamin (Lavand'homme and Roelant, 2009), Pregabalin (Olesen et al., 2013) and Oxycodon (Eisenberg et al., 2010). It is argued that this fact relates to the ability of these substances to reduce spinal synaptic fascilitation (Yarnitsky, 2015). According to numerous animal experiments (mostly conducted in rats), the same can be assumed for acupuncture (Zhang et al., 2014). In particular, electroacupuncture was found to mediate its analgesic effect in different animal pain models (cancer pain, muscle pain and inflammatory pain) through reducing spinal substance P concentration (SP) (Lee et al., 2009; Hsieh et al., 2014) and counteracting the upregulation of the neurokinin receptor 1 (NK1) (Zhang et al., 2005) as well as SP-NK1 downstream targets such as phosphatidylinositol 3-kinase and Akt (Kim et al., 2012). Additionally, reduced hyperalgesia after electroacupuncture was associated with lower upregulation of NGF in a diabetes model (Manni et al., 2011) and decreased NMDA receptor phosphorylation (Tian et al., 2008) as well as lower density of the NMDA subunit (NR2B) in a model of irritable bowel syndrome (Liu et al., 2016). Further acupuncture mechanisms counteracting spinal synaptic facilitation seem to include inhibition of glia cell activation (Lin et al., 2016). In line with this, electroacupuncture reduced spinal concentrations of interleukin-1β in a bone-cancer model (Zhang et al., 2007) and of nitric oxide in a neuropathic pain model (Cha et al., 2010).

Investigations of acupuncture effects on TS in humans that would provide further evidence of its spinal anti-fascilitatory effects are sparse. According to one trial electroacupuncture, but not manual acupuncture decreased TS in healthy volunteers (Zheng et al., 2010), while four studies, two also in healthy volunteers (Lang et al., 2010; Baeumler et al., 2015), one in whip-lash patients (Tobbackx et al., 2013) and one recent trial in patients with chronic low back pain (Leite et al., 2018) found neither electroacupuncture nor manual acupuncture to affect TS. Two main legitimate objections have been raised regarding these conflicting results (Kong et al., 2013). First, except for one trial, effects of a single acupuncture treatment were assessed. However, a clinically relevant reduction of spinal synaptic facilitation would rather be expected after a series of acupuncture sessions. Second, wind-up in healthy volunteers is already low and, thus, may hardly be affected by regulatory interventions such as acupuncture. In addition, methodological differences between studies such as the nociceptive stimulus used to elicit TS and the use of absolute pain scores elicited by repetitive stimuli instead of the ratio between single and repetitive stimuli accounting for overall pain sensitivity may give rise to further source of bias.

High TS at the control site was positively associated with reduced pain intensity after the first acupuncture treatment as indicated by both logistic regression and by the cumulative response function. Additionally, high TS at the control site was associated with subjective pain relief as evaluated by the questionnaire. In contrast, the association of high TS at the most painful body site was only associated with the subjective experience of an immediate pain relief of over 50%. This indicates that propagated synaptic facilitation throughout the spinal cord, e.g., through volume transmission (Zieglgänsberger, 2009), as a sign of advanced central sensitization is more important for the prediction of immediate analgesic acupuncture effects than primary synaptic facilitation. External validity of these results can be assumed high, since no specific selection criteria were applied. The cut-off definition for a high TS as a WUR of over 2.5 applied in our study may also be useful in future trials, as it was confirmed by an ROC-analysis and it matches the population mean established in healthy controls which appears constant over various body sites (Rolke et al., 2006; Puta et al., 2013; Pfau et al., 2014).

Few studies had previously assessed sensory signs as evaluated by QST as predictors for the analgesic effect of acupuncture. Two trials from the same research group identified a low pressure pain threshold at the thumbnail to predict a good response to sham-acupuncture (needling at non-acupuncture points or non-penetrating sham) in fibromyalgia (Harte et al., 2013; Zucker et al., 2017). In their second trial (Zucker et al., 2017), conversely, a low pressure pain threshold predicted a non-response verum acupuncture and strong needle sensations even caused an increase in clinical pain. In addition, a low electrical pain threshold in a pain-free body site has also been found to be lower in electroacupuncture non-responders than responders among patients suffering from painful burn scars (Cuignet et al., 2015). Authors discuss that the strength of needle stimulation needs to be adapted to the patients' sensitivity in order to maximize treatment effects and to avoid unfavorable outcomes. As treatments in our trial were individually tailored, it can be assumed that bias caused by needle stimulation exceeding the patients' comfort zone was minimal.

We found no association of TS with the patients' subjective appraisal of the contribution of acupuncture to overall pain relief during the 4-week multimodal pain treatment program. However, it needs to be emphasized that our study was not designed to confirm predictors for pain reduction after a series of acupuncture treatments. The appraisal of the contribution of acupuncture to overall pain reduction is likely to be affected by treatment success of the whole multimodal pain program involving a variety of therapy modalities. Therefore, the prediction of the long-term effects of acupuncture by TS and other somatosensory thresholds remain subject of further investigations.

### Confounding by Age

Age confounded the association of high TS at the control site with the immediate analgesic acupuncture effect and age-adjusted ORs were larger than crude OR. Collinearity was ruled out according to the VIF of the respective logistic regression models. Thus, one can conclude that older age was positively associated with response to acupuncture and marginally more frequent in patients with high TS at the control site. The latter is in line with previous findings indicating that age does not substantially influence heat evoked TS in fibromyalgia patients (Staud et al., 2008) and pressure evoked TS in patients suffering from knee osteoarthritis (Petersen et al., 2017). In contrast, the relation of age with TS remains elusive in healthy adults in which heat evoked TS was found to be positively associated with age (Edwards and Fillingim, 2001) while pin-prick evoked TS seems age-independent (Magerl et al., 2010).

It can only be speculated why the chance to experience an immediate analgesic effect through acupuncture increased by 6–10% with every year of age. We are only aware of previous studies that assessed the influence of age on long-term pain relief achieved by acupuncture. A reanalysis of the large German acupuncture trials revealed that pain reduction after a series of verum- as well as after a series of sham-acupuncture was weakly associated with younger age (Witt et al., 2011). A more recent qualitative patient assessment of acupuncture effects revealed no association with age (Cramer et al., 2015). Our finding of a more pronounced immediate acupuncture effect in older patients might be explained by the fact that sensory stimulation, such as applied during needling, might have stronger impact on older patients. Sensory perception is known to decrease with age (Magerl et al., 2010), but was shown to be more efficiently modulated by conditioning stimuli in older than in younger subjects (Naugle et al., 2015).

### No Association Between Vibration Detection Threshold and Acupuncture Effects

The hypothesis that poor Aβ-fiber function indicated by a loss in vibration detection would predict a poor acupuncture response was not confirmed. However, the large variance in VDT data particularly at the pain sites hampers interpretability of this finding. It is known that the VDT varies largely between body sites (Rolke et al., 2006). Our results once again suggest that a meaningful interpretation of the VDT in clinical practice would require reference values for a large variety of body sites. These are not yet established. To fully reject or approve our hypothesis, patient populations with consistent pain sites might be assessed. It should be considered that supra-spinal and local acupuncture mechanisms might compensate the lack of segmental inhibition related to poor Aβ-fiber function. Consequently, poor VDT might still be associated with immediate analgesic effects of a purely segmental needling regimen. An association between long-term acupuncture effects and the VDT in turn appears unlikely, as segmental effects inducing long-term depression of spinal synaptic pain transmission rely on the activation of Aδ- rather than Aβ-fibers. Furthermore, a loss of mechanical detection that has been described in several also non-neuropathic chronic pain conditions (Geber et al., 2008; Westermann et al., 2011; Puta et al., 2013) might not necessarily be caused by a loss in Aβ-fiber function but rather by a presynaptic inhibition linked to the constant nociceptive input (Janig and Zimmermann, 1971). If the latter hypothesis proves true, a reduced activation of the nociceptive system after a successful acupuncture treatment would also be reflected by an increased ability to detect mechanical stimuli. Future studies on the effectiveness of acupuncture in neuropathic pain conditions might explore this hypothesis.

## Limitations

A source of bias in this study is that recruitment and data collection were integrated into the course of a multimodal pain treatment program. Thus, overall treatment satisfaction might have influenced patients' rating of acupuncture effects. Social desirability bias (Furnham, 1986) might have been caused by the fact that baseline and follow-up examinations were performed by the same examiner. However, evaluations of the reduction in pain intensity after the first acupuncture treatment were free from such bias, as acupuncture was always applied as the first treatment modality on the first day of the multimodal pain program. Furthermore, patients took their usual pain medication. However, it seem unlikely that the intake of analgesics has affected the results. First, short-term drug level changes were avoided as stable pain medication intake is a precondition to take part in the multimodal pain treatment program performed at the study center. Second, the use of analgesics was neither a confounder nor an effect modifier for the associations under investigation, nor was it confirmed to be an independent predictor for pain relief after acupuncture in the multivariate analysis. Additionally, excluding patients on pain medication would have rendered our sample not representative for the average population of chronic pain patients that an acupuncturist encounters in daily clinical practice.

Additionally, it is known that multiple testing might lead to false inferences in observational studies, as they require adjustment for various covariates which automatically increases the number of hypotheses tested (Patel and Ioannidis, 2014; Gruber and Tchetgen, 2016). Consensus on strategies for a meaningful p-value adjustment has not been found. However, Bonferroni correction of each predictor hypothesis test for the four outcome parameters (VRS pain score, subjective immediate and overall pain relief and effect on wellbeing) would results a critical p-value of 0.0125. Under this assumption, associations between the WUR at the control site but not the pain site would remain statistically significant; supporting the notion that WUR in a pain free control site is a more promising predictor for response to acupuncture. It is argued that hypothesis driven analyses are best to omit bias arising from multiple testing. The design of this study is based on hypotheses driven by clinical and neurophysiological considerations and previous observations. Nevertheless, statistical analyses were performed in an exploratory manner to optimally explain variation in the data. Thus, these results require replication by future confirmatory research and validation of the proposed predictor models in an independent population.

### Perspectives

Our results are an important step toward an individualized pain therapy in regard to non-pharmacological treatments based on sensory profiles. Expenditure of resources to explore TS in chronic pain patients as a predictor also for long-term acupuncture effects is now justified. In addition, further research is needed to explore whether effectiveness of acupuncture in certain pain conditions such as neuropathic pain, low back pain etc. can be predicted by strong signs of central sensitization. Another sensory sign of interest in this regard might be the conditioned pain modulation (CPM) indicating descending pain control. Acupuncture has been shown to activate descending pain control mechanisms, but it has not yet been investigated whether CPM predicts acupuncture effects, or whether a lack in CPM can be reestablished by continuous acupuncture treatment.

## Conclusion

Strong central sensitization reflected by high TS, in particular at pain free control sites, seems to predict the immediate analgesic response to acupuncture. Clinically spoken, acupuncture could be especially effective in highly sensitized chronic pain patients.

## Ethics Statement

Study conduct was in accordance with the Declaration of Helsinki formulated by the World Medical Association [updated version, Seoul (The World Medical Association, 2008)] and was approved by the ethics committee of the Medical Faculty of the Ludwig-Maximilians University of Munich. Written informed consent was obtained from all participants. Withdrawal from study procedures was open to participants at any time. In case of withdrawal all data recorded in the course of the study were deleted. Data handling was performed in pseudonymous form and followed the German data-protection act.

## Author Contributions

PB, DI, and PC conceptualized the study design, contributed to interpretation of the results and drafted the manuscript. PB was in charge of patient recruitment, data-acquisition, and data-analysis.

### Conflict of Interest Statement

PB was presented with the Young Scientists Award (sponsored by 3B-Scientific) for an oral presentation of the results presented in this manuscript at the congress of the International Council for Acupuncture and Related Techniques (ICMART) in June 2017. PB and DI receive honorarium and travel costs from non-profit academic organizations, physician chambers and universities for teaching and lecturing. The remaining author declares that the research was conducted in the absence of any commercial or financial relationships that could be construed as a potential conflict of interest.
